# Endobronchial Leiomyoma: A case report with cryoprobe extraction and review of literature

**DOI:** 10.1016/j.rmcr.2021.101467

**Published:** 2021-07-06

**Authors:** Hasnain Bawaadam, Nathaniel Ivanick, Ihab AlShelli, Ganesh Krishna

**Affiliations:** a Department of Medicine, Division of Pulmonary and Critical Care, University of California San Francisco, San Francisco, CA, USA; b Department of Thoracic Surgery, Roswell Park Comprehensive Cancer Center, Buffalo, NY, USA; c Department of Pulmonary Medicine, Cleveland Clinic, Weston, FL, USA; d Department of Medicine, Division of Pulmonary and Critical Care, Palo Alto Medical Foundation, Palo Alto, CA, USA

**Keywords:** None

## Abstract

Large airway tumors are uncommon, accounting for about 0.6% of all pulmonary tumors [[1], [2], [3]]. The majority of these tumors (80–90%) are malignant, represented primarily by squamous cell carcinoma and adenoid cystic carcinoma [2,4]. Benign central airway tumors are less common and are generally comprised of hamartomas and papillomas. Tracheobronchial leiomyomas are exceedingly rare, representing only about 0.6% of all benign lung neoplasms [3]. We report here on a case of primary endobronchial leiomyoma without uterine involvement treated successfully with cryoresection with excellent outcome.

## 1 Case report

**Fig. 1:**
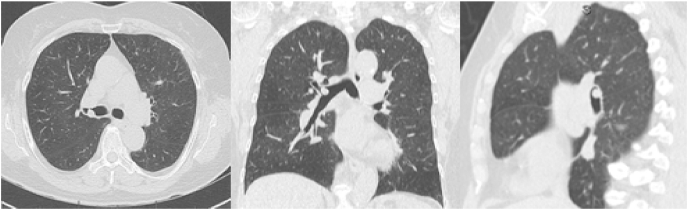
Axial, Coronal and Sagittal Imaging showing the Leiomyoma in the right mainstem bronchus. Note the origin from the posterior (wall).

A 72-year-old Asian female smoker underwent her initial lung cancer screening computed tomography (CT) scan, which demonstrated an 8mm nodule in the right mainstem bronchus (Fig. 1). The patient denied symptoms of cough, dyspnea, fevers, or weight loss. Her personal medical history and family history were otherwise unremarkable.

**Fig. 2:**
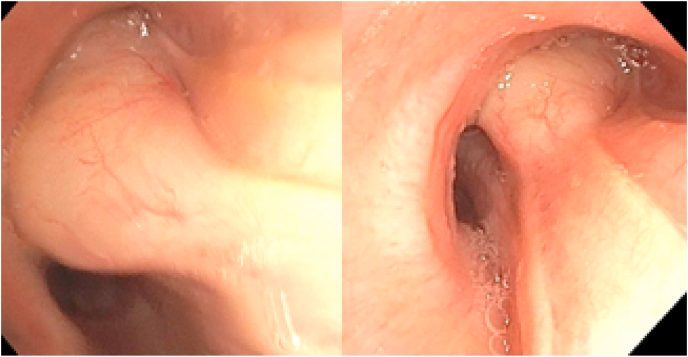
Leiomyoma in the right mainstem bronchus as seen during flexible bronchoscopy.

On flexible bronchoscopy a wide-based mass in the right mainstem bronchus just proximal to the takeoff of the right upper lobe was identified (Fig. 2). There was over 90% luminal obstruction of the right upper lobe bronchus. With the use of the 2.2mm cryoprobe, the lesion was extracted with repeated activations of the cryoprobe. Cryoprobe therapy deals with the destruction of tissue through the cytotoxic effects of freezing with nitrous oxide (N2O) delivered through a cold-resistant metal catheter. Hemostasis at the base of the tumor was achieved using Argon Plasma Coagulation (APC) which is a noncontact method that allows for controlled electrocoagulation via high-frequency monopolar energy to be delivered to the tissue through an ionized gas (argon plasma). At the completion of the procedure, the right upper lobe bronchus was open, and a careful airway inspection revealed no other endobronchial lesions.

**Fig. 3:**
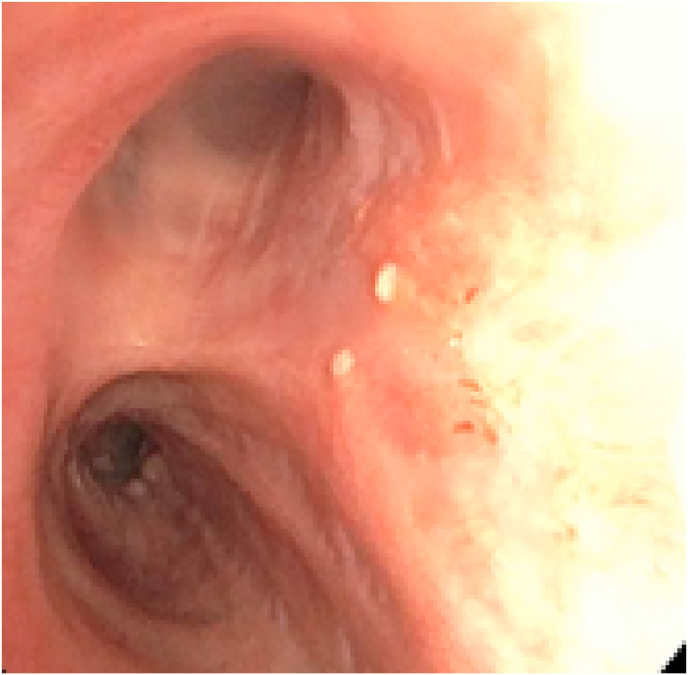
Right middle lobe takeoff noted after successful cryosurgical resection 6 months later.

**Fig. 4:**
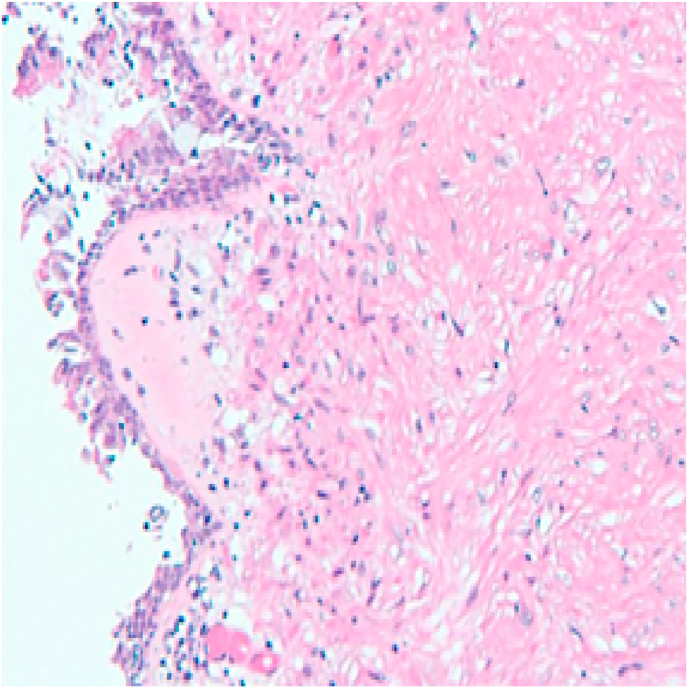
Hematoxylin and Eosin Stain (Original Magnification ×10).

**Fig. 5:**
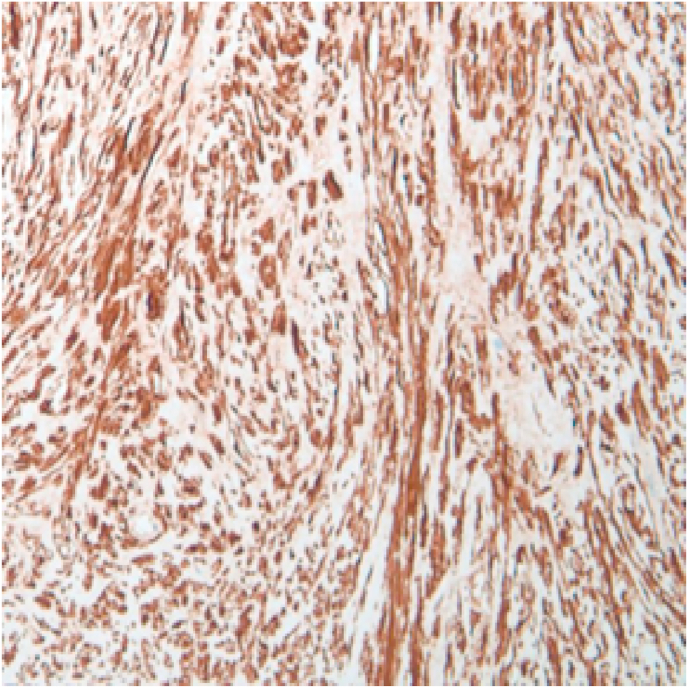
Smooth Muscle Cells Staining positive for Actin and Desmin (Original Magnification ×10).

Pathology review of these specimens yielded a diagnosis of tracheobronchial leiomyoma (Fig. 4). This was confirmed with immunohistochemical stain for smooth muscle actin and desmin (Fig. 5). After discovery, the patient received a CT scan of the abdomen and pelvis as well as a transvaginal ultrasound of the uterus that did not show any uterine fibroids. On surveillance bronchoscopy 6 months later she was noted to have two small and non-obstructive airway nodules on the posterior bronchial surface in the area of the previous tumor base (Fig. 3). These were cryoablated using a 1.8mm cryoprobe and excised with the CoreCath™ catheter electrocautery device that achieves both dissection as well as hemostasis of the targeted lesion. Specimen was not sent to pathology during the second procedure.

## 2 Discussion

Benign pulmonary tumors are uncommon with a reported incidence of 4% among all surgically excised tumors [1]. In general, benign tumors of the tracheobronchial tree arise from mesenchymal structures, whereas malignant tumors arise from the epithelium or salivary glands [4]. Primary endobronchial leiomyomas represent only 2% of benign tracheal tumors, making them exceedingly rare [4]. They can be found anywhere along the tracheobronchial tree or within the lung parenchyma. Leiomyomas arise from the smooth muscle cells that constitute the mesenchymal layer, or from smooth muscles of the bronchioles or blood vessels [1,3]. Leiomyomas can be found as solitary lesions, as part of a diffuse or multifocal leiomyomatosis or benign metastasizing leiomyomas associated with primary uterine leiomyoma [5]. Airway leiomyomas have equal prevalence in males and females and are typically identified in middle age whereas pulmonary parenchymal leiomyomas have a female to male ratio of 2:1 with no age predilection [3].

Histologically, leiomyomas are tumors of mesenchymal origin, composed of proliferating spindle cells, typically with low mitotic activity, similar to other spindle cell tumors such as fibroma, neurofibroma, and schwannoma. Unlike the aforementioned tumors, immunohistochemical staining is strongly positive for smooth muscle actin and desmin in leiomyomas [1,3]. Histologically, airway leiomyomas demonstrate bundles of smooth muscle cells with minimal fibrous and vascular components, whereas the latter is more common in pulmonary parenchymal leiomyomas.

As with most tracheobronchial tumors, the type and degree of symptoms reflect the tumor's size, location in the airway and degree of airway obstruction. Symptoms encountered are nonspecific and may include dyspnea, cough, wheezing, post-obstructive pneumonia, atelectasis, bronchiectasis, and hemoptysis. Pneumothoraces have been described as caused by air trapping and resultant pneumothorax [6]. The presence of intermittent dyspnea and wheezing has been confused with asthma. Focal inspiratory wheezing should suggest an alternative diagnosis to asthma [7]. It is interesting that our patient did not manifest more symptoms, considering the degree of airway obstruction. This may reflect the upper lobe location allowing for adequate mucociliary clearance, as well as its location just above the bronchus intermedius, allowing for ventilation of the majority of the right lung, if not the upper lobe. As in our patient, leiomyomas are typically round, oval or lobulated, often with a wide base.

On cross sectional imaging, leiomyomas appear homogenous with low attenuation (25–46 Hounsfield Units (HU) on unenhanced CT and 46–85 HU on enhanced CT [8,9]. Calcification is a rare finding with only several instances reported [10,11]. Recently, these tumors have even been reported to cause an intense FDG uptake on PET/CT mimicking malignancy [12].

The treatment of tracheobronchial leiomyoma is surgical resection due to the tendency to grow and cause airway obstruction. Primary tracheobronchial leiomyomas have a very low potential for malignant transformation, although this has been described in benign metastasizing leiomyoma [13,14]. Resection is expected to offer cure, particularly for primary tracheobronchial leiomyomas. Before bronchoscopic resection techniques were widely available, most of these tumors were treated surgically with lobectomy, sleeve resection, or rarely, pneumonectomy. Other techniques include bronchotomy with local resection, or bronchoplastic resection [15]. With advances in bronchoscopic based therapies, most central tracheobronchial leiomyomas can be treated bronchoscopically with less comorbidity. Heat based modalities including electrocautery, argon plasma coagulation, and laser with and without mechanical debulking have been used to excise these tumors [10,[16], [17], [18]] and bronchoscopic cryosurgery for leiomyoma has been described previously in the literature as well [10,19]. Although, cold based debulking options are better than heat based options due to decreased incidence of cicatrization and granulation tissue formation, a combined modality approach is often used. We used cryo-extraction as the predominant mode of debulking and used APC for coagulation. One important predictor of successful resection is the width of the base, with wider lesions predicting higher risk of recurrence. Patients should be monitored at regular intervals to ensure no recurrence has occurred.

## 3 Conclusion

Tracheobronchial leiomyomas are very rare benign tumors of the airways and lung parenchyma. When identified, bronchoscopic resection using hot or cold ablative modalities offer a great chance of success, with minimal morbidity to the patient and the incidence of recurrence not well established. We report on a case of right mainstem bronchus leiomyoma successfully resected with cryoprobe extraction and APC for hemostasis.

## Declaration of competing interest

No conflict of interest declared.Appropriate written informed consent was obtained for publication of this case report and accompanying images.
